# Macrolide resistance in Staphylococcus from COVID-19 patients in Santander

**DOI:** 10.15649/cuidarte.4924

**Published:** 2025-12-17

**Authors:** Michael J. Santos-Angarita, Monica Y. Arias Guerrero, Andrea J. Parada-Diaz, Natalia A. Bravo Granados, Nadia C. Alfonso Vargas, Juanita Trejos-Suárez

**Affiliations:** 1 Universidad de Santander, Faculty of Medical and Health Sciences, MASIRA Research Institute. Bucaramanga, Colombia. E-mail: buc21171003@mail.udes.edu.co Universidad de Santander Bucaramanga Colombia buc21171003@mail.udes.edu.co; 2 Universidad de Santander, Faculty of Medical and Health Sciences, MASIRA Research Institute. Bucaramanga, Colombia. E-mail: moni.arias@mail.udes.edu.co Universidad de Santander Bucaramanga Colombia moni.arias@mail.udes.edu.co; 3 Universidad de Santander, Faculty of Medical and Health Sciences, MASIRA Research Institute. Bucaramanga, Colombia. E-mail: and.parada@mail.udes.edu.co Universidad de Santander Bucaramanga Colombia and.parada@mail.udes.edu.co; 4 Universidad de Santander, CBUDES Biological Collection. Bucaramanga, Colombia. E mail: cbudes@udes.edu.co Universidad de Santander Bucaramanga Colombia cbudes@udes.edu.co; 5 Universidad de Boyacá, Faculty of Health Sciences, Bacteriology and Clinical Laboratory Program. Tunja, Colombia. E-mail: ncalfonso@uniboyaca.edu.co Universidad de Boyacá Boyacá Colombia ncalfonso@uniboyaca.edu.co; 6 Universidad de Santander, Faculty of Medical and Health Sciences, MASIRA Research Institute. Bucaramanga, Colombia. E-mail: juanita.trejos@udes.edu.co Universidad de Santander Bucaramanga Colombia juanita.trejos@udes.edu.co

**Keywords:** Co-infection, COVID-19, Staphylococcus, Macrolides, Antibiotic Resistance, Coinfección, COVID-19, Staphylococcus, Macrólidos, Resistencia a Antibióticos, Coinfecção, COVID-19, Staphylococcus, Macrolídeos, Resistência a Antibióticos

## Abstract

**Introduction:**

Intensive use of macrolides, such as azithromycin, during the COVID-19 pandemic has facilitated the development of antimicrobial resistance in Gram-positive bacteria through multiple resistance mechanisms, including ribosomal RNA modification, efflux pumps, and enzymatic inactivation.

**Objective:**

To describe the prevalence of resistance genes in bacteria isolated from COVID-19 patients in Santander, Colombia.

**Materials and Methods:**

A descriptive study was conducted on 112 stored samples from nasopharyngeal and oropharyngeal swabs and tracheal aspirates collected from hospitalized COVID-19 patients in 2020, from which 48 Gram-positive strains were isolated. Macrolide resistance and the presence of the ermA, ermB, ermT, and mef(A/E) genes were evaluated through phenotypic and molecular tests.

**Results:**

Staphylococcus aureus was the most prevalent species at 58.33% (28), followed by Staphylococcus epidermidis at 31.25% (15). A total of 47.92% (23) of the strains showed phenotypic resistance to azithromycin, and 81.25% (39) displayed genotypic resistance, with ermB being the most prevalent at 58.33% (28) and ermT at 45.83% (22), with no detection of mef(A/E).

**Discussion:**

These findings reveal a high prevalence of macrolide resistance, which may be related to the extensive use of these antibiotics during the pandemic.

**Conclusion:**

The increase in macrolide resistance among Gram-positive bacteria represents a critical public health challenge, especially in the context of pandemics. These results underscore the urgent need to implement control measures in antibiotic use.

## Resumen

**Introducción:**

El uso intensivo de macrólidos, como la azitromicina, durante la pandemia de COVID-19 ha favorecido el desarrollo de resistencia antimicrobiana en bacterias Grampositivas a través de múltiples mecanismos de resistencia, como la modificación del ARN ribosomal, bombas de expulsión e inactivación enzimática.

**Objetivo:**

Describir la prevalencia de genes de resistencia en bacterias aisladas de pacientes con COVID-19 en Santander, Colombia.

**Materiales y métodos:**

Se realizó un estudio descriptivo de 112 muestras almacenadas de hisopados nasofaríngeos, orofaríngeos y aspirados traqueales de pacientes hospitalizados con COVID-19 en 2020, de las cuales se aislaron 48 cepas Grampositivas. La resistencia a macrólidos y la presencia de los genes ermA, ermB, ermT y mef(A/E) se evaluaron mediante pruebas fenotípicas y moleculares.

**Resultados:**

Staphylococcus aureus fue la especie más prevalente con un 58,33% (28), seguida de Staphylococcus epidermidis con un 31,25% (15). Un 47,92% (23) de las cepas mostró resistencia fenotípica a la azitromicina, y un 81,25% (39) resistencia genotípica, con predominancia de ermB con el 58,33% (28) y ermT con el 45,83% (22), sin detección de mef(A/E).

**Discusión:**

Estos hallazgos muestran una alta prevalencia de resistencia a macrólidos, lo cual puede estar relacionado con el uso extensivo de estos antibióticos durante la pandemia.

**Conclusiones:**

El aumento en la resistencia a macrólidos en bacterias Grampositivas representa un reto crítico para la salud pública, especialmente en el contexto de pandemias. Estos resultados subrayan la necesidad urgente de implementar medidas de control en el uso de antibióticos.

## Resumo

**Introdução:**

O uso intensivo de macrolídeos, como a azitromicina, durante a pandemia de COVID-19 favoreceu o desenvolvimento de resistência antimicrobiana em bactérias Gram-positivas por meio de múltiplos mecanismos de resistência, como modificação do RNA ribossômico, bombas de efluxo e inativação enzimática.

**Objetivo:**

Descrever a prevalência de genes de resistência em bactérias isoladas de pacientes com COVID-19 em Santander, Colômbia.

**Materiais e métodos:**

Foi realizado um estudo descritivo com 112 amostras de cotonetes nasofaríngeo e orofaríngeo armazenadas e aspirados traqueais de pacientes hospitalizados com COVID-19 em 2020, dos quais 48 cepas Gram- positivas foram isoladas. A resistência a macrolídeos e a presença dos genes ermA, ermB, ermT y mef(A/E) foram avaliadas por testes fenotípicos e moleculares.

**Resultados:**

Staphylococcus aureus foi a espécie mais prevalente com 58,33% (28), seguido por Staphylococcus epidermidis com 31,25% (15). 47,92% (23) das cepas apresentaram resistência fenotípica à azitromicina e 81,25% (39) resistência genotípica, com predominância de ermB com 58,33% (28) e ermT com 45,83% (22), sem detecção de mef (A/E).

**Discussão:**

Esses achados mostram uma alta prevalência de resistência a macrolídeos, o que pode estar relacionado ao uso extensivo desses antibióticos durante a pandemia.

**Conclusões:**

O aumento da resistência a macrolídeos em bactérias Gram-positivas representa um desafio crítico à saúde pública, especialmente no contexto de pandemias. Esses resultados ressaltam a necessidade urgente de implementar medidas de controle de antibióticos.

## Introduction

Macrolides, such as azithromycin, erythromycin, and clarithromycin, are essential antibiotics in the treatment of bacterial infections. These hydrophobic compounds exert a bacteriostatic effect by blocking protein synthesis through their binding to the peptidyl transferase of 23S ribosomal ribonucleic acid (rRNA) within the 50S subunit of the bacterial ribosome^1^,^2^.

During the COVID-19 pandemic, the use of macrolides increased considerably, particularly azithromycin, as part of prophylactic and therapeutic strategies. In many cases, this use was inappropriate and contributed to the global increase in antimicrobial resistance^3^,^4^. In Latin America, high rates of antibiotic self-medication have been reported, hindering the clinical management of many of these patients and favoring the development of resistance in pathogens prevalent in the region^5^-^7^.

In Gram-positive bacteria, resistance to macrolides develops mainly through three mechanisms: 1) modification of the target site in 23S rRNA, 2) active efflux mediated by efflux pumps, and 3) enzymatic inactivation, although the latter is less frequent^8^,^9^. These mechanisms, which have been associated with genes such as ermA, ermB, ermT, and mef(A/E), limit treatment options and represent a challenge in hospital settings, particularly for vulnerable patients.

International studies have identified a high prevalence of macrolide resistance genes in Staphylococcus aureus and Streptococcus pyogenes, as observed in Peru and Chile^10^-^12^. However, in Colombia, there are few epidemiological data on this subject, which hinders the development of effective control strategies. This study aimed to determine the prevalence of the ermA, ermB, ermT, and mef(A/E) genes in Gram-positive strains isolated from hospitalized patients with COVID-19 in Santander, Colombia. These findings will contribute to a better understanding of the local resistance landscape and support the development of more effective surveillance and control strategies in the context of public health.

## Materials and Methods

**Sample Collection and Selection: **A descriptive cross-sectional study was conducted, in which 112 samples were randomly selected from a total of 2,072 stored as part of the COVID-19 epidemiological surveillance program, collected between July 2020 and January 2021 in various municipalities of the Department of Santander, Colombia. The samples—which included nasopharyngeal swabs, oropharyngeal swabs, and tracheal aspirates—were obtained from hospitalized patients of all ages and sexes for COVID-19 diagnosis, and the collection was carried out under the Special Cooperation Agreement No. 065 between the Instituto Nacional de Salud (INS) of Colombia and the Universidad de Santander.

For analysis, cultures showing significant bacterial growth were selected, as evidenced by growth in at least two quadrants of the agar plate, in which the primary pathogen predominated over accompanying microbiota. Patients under antimicrobial treatment or with prior antibiotic use before COVID-19 diagnosis were not excluded, as this information was not available within the epidemiological surveillance process.

The sample size was calculated using IBM® SPSS® Statistics version 26.0, with a 95% confidence level, a 5% margin of error, and a 31% prevalence, based on bacterial co-infection data reported by Buehrle et al. (2020)^13^.

**Microbiological Cultures and Isolates: **Swabs or aspirates were directly cultured on 5% Blood Agar and Chocolate Agar, incubated in an atmosphere enriched with 5% CO₂, and on Mannitol Salt Agar under aerobic conditions, at 37°C for 24–48 hours. To ensure growth quality, reference strains were inoculated as positive controls to guarantee the performance of the media and incubation conditions, verifying that optimal growth conditions were met. Culture recovery was considered successful when bacterial growth covered at least two quadrants of the plate, showing the pathogen’ predominance over the background microbiota. Each recovered strain underwent quality control to assess purity and viability, including Gram staining and subculturing on 5% Blood Agar to obtain an axenic culture, incubated for 24 hours at 37°C.

**Identification of Bacterial Strains:** The phenotypic identification of Gram-positive cocci was carried out using biochemical tests (catalase, coagulase, and Rapid Staph Plus System) and morphological characteristics observed on the Gram stain. Genotypic identification focused on the detection of specific Staphylococcus genes, as no applicable results were obtained for Streptococcus.

For genotypic identification, deoxyribonucleic acid (DNA) was extracted by thermal lysis, with a pretreatment using 10 mM EDTA (pH 8.0) and 1 mg/mL Proteinase K, following the standardization of the protocol with reference control strains (S. aureus subsp. aureus ATCC 25923 and S. epidermidis ATCC 35984). The quality and concentration of the extracted DNA were evaluated using a NanoDrop™ 2000c spectrophotometer (Thermo Fisher Scientific, USA).

The molecular characterization of bacterial strains was performed by multiplex polymerase chain reaction (PCR) using the nuc genes, which encode thermonucleases widely conserved among the main Staphylococcus species^14^. PCR reactions were prepared in a final volume of 50 μL containing Taq 2X PCR MasterMix (Applied Biological Materials Inc., Canada), 0.5 µM of each primer, 10 ng of DNA, and nuclease-free water.

Amplified DNA fragments were analyzed by electrophoresis on a 1.2% agarose gel containing the Midori Green Advance™ intercalating dye (Nippon Genetics Europe GmbH, Germany), at 90 V for 70 minutes in 1X TAE buffer (Tris-Acetate-EDTA).

**Phenotypic Resistance to Azithromycin:** Phenotypic resistance was evaluated using the Kirby– Bauer disk diffusion method on Mueller–Hinton agar (Thermo Scientific™ Oxoid™, USA), employing 15 µg azithromycin disks (AZM – Thermo Scientific™ Oxoid™, USA), following the recommendations of the CLSI M100-Ed34:2024 guideline^15^. This method was chosen due to its widespread use in epidemiological surveillance studies and its recommendation for antimicrobial susceptibility testing. Although it is recognized that this method may have lower susceptibility compared with techniques such as microdilution, its standardization, low cost, and reproducibility make it suitable for exploratory studies such as this one. Plates were incubated at 37°C for 18–24 hours before measuring inhibition zone diameters. The interpretative breakpoints for inhibition zones were as follows: susceptible (≥15 mm), intermediate susceptibility (14–17 mm), and resistant (≤8 mm).

**Molecular Characterization of Macrolide Resistance Genes:** Characterization was performed by PCR following previously described, unmodified protocols for ermA and ermB^16^, ermT^17^, and mef(A/E)^18^. PCR reaction mixtures were prepared in a final volume of 50 μL, containing Taq 2X PCR MasterMix (Applied Biological Materials Inc., Canada), 0.5 µM of each primer, 10 ng of DNA, and nuclease-free water. The amplified fragments were analyzed by electrophoresis on a 1.2% agarose gel containing the Midori Green Advance™ intercalating dye (Nippon Genetics Europe GmbH, Germany), at 90 V for 70 minutes in 1X TAE buffer (Tris-Acetate-EDTA).

**Sequencing and Bioinformatic Analysis:** The amplified genes were sent for sequencing using the express-seq method at Gencell Pharma©, employing the same primers used in the PCR reactions to ensure sequence specificity. The resulting reads, in .ab1 format (Forward and Reverse), underwent an initial quality control using FinchTV (Geospiza, Inc., USA), evaluating the clarity and precision of fluorescence peaks and verifying the sequence integrity. This preliminary inspection allowed the identification of potential sequencing artifacts, ensuring that only high-quality reads proceeded to subsequent analysis. Subsequently, on the Galaxy Europe server (https://usegalaxy.eu/), the sequences in .ab1 format were converted to FASTQ format, and quality control tools such as FastQC were applied to examine per-base quality scores (Q20 and Q30), GC content distribution, presence of adapters, and average read length. To obtain the complete amplicon sequence, Forward and Reverse reads were assembled using CAP3, generating a highly accurate contig representing the amplified fragment. Manual inspection in FinchTV of the overlapping region between Forward and Reverse reads made it possible to rule out assembly discrepancies.

The assembled sequence was then analyzed using BLASTn^19^ to detect specific previously described variants, with e-value and minimum coverage parameters configured to ensure accurate homology identification. To characterize resistance genes, the CARD^20^, ResFinder 4.0^21^,^22^, and ARG-ANNOT^23^ databases were used, enabling a comprehensive evaluation of resistance variants and mechanisms associated with mutations in the genes of interest.

**Statistical Analysis:** Fisher’s exact test or Pearson’s chi-square test was used to assess the association between phenotypic and genotypic resistance, selecting the appropriate test according to the frequencies in the contingency tables, particularly in cases of low frequencies. In addition, the frequency distributions of resistance genes were calculated using IBM® SPSS® Statistics, version 26.0.

**Ethical Considerations:** This study did not involve direct experimentation on humans or animals. The bacterial samples used were obtained from nasopharyngeal and oropharyngeal swabs, and from tracheal aspirates collected from hospitalized patients during 2020 as part of the COVID-19 epidemiological surveillance program. These samples were stored at −80°C at the Universidad de Santander in accordance with biosafety standards and with authorization from the Instituto Nacional de Salud (INS) of Colombia, under Special Cooperation Agreement No. 065, to be used for research on bacterial co-infections. Since the samples were obtained through health surveillance activities, informed consent from patients was not required, in accordance with national and international research ethics regulations, including the CIOMS guidelines. The research protocol was approved by the Bioethics Committee of the Universidad de Santander, as recorded in Minutes No. 006 dated March 8, 2022, ensuring compliance with the bioethical principles of autonomy, beneficence, non- maleficence, and justice.

The isolated strains were deposited in the Biological Collection of Microorganisms of the Universidad de Santander, CBUDES (RNC: 280; WDCM 1264). The data supporting the findings of this study are available in a public and institutional repository on Figshare^24^, preserving participant confidentiality through an anonymized version of the dataset. This repository includes Excel files and other relevant documents, facilitating the review and detailed analysis of the results.

## Results

**Figure 1:**
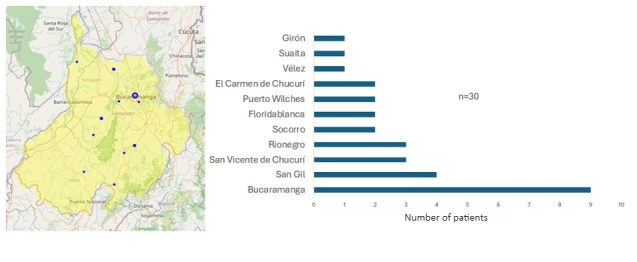
Geographical distribution and frequency of hospitalized COVID-19 patients in Santander, Colombia.

From the 112 samples analyzed in this study, 48 Gram-positive cocci were isolated from 30 Colombian patients who were hospitalized and diagnosed with COVID-19 in various municipalities of the Department of Santander, Colombia. The distribution and frequency of patients by hospitalization municipality are shown in Figure 1. The median patient age was 51.5 years (95% CI: 39.66–56.26), with a minimum age of 6 years and a maximum of 87 years. In terms of survival status, 90.00% (n=27) of the patients were alive, 6.66% (n=2) had died, and 3.33% (n=1) had an unknown vital status.

The map on the left shows the location of each municipality with patients from whom Gram- positive bacteria were isolated, while the bar chart on the right indicates the number of patients per municipality.

**Sample Collection and Microbial Characterization**

**Figure 2:**
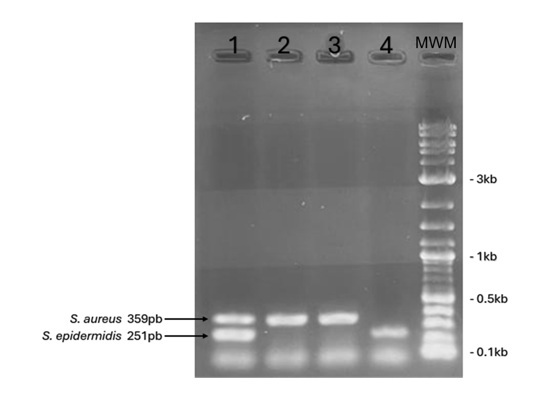
Characterization of S. aureus and S. epidermidis by PCR through detection of the nuc gene.

From the 112 samples analyzed, 48 Gram-positive cocci were isolated from 30 patients, distributed as follows: S. aureus 58.33% (n=28), S. epidermidis 31.25% (n=15); other isolated Staphylococcus species included S. chromogenes 2.08% (n=1), S. saprophyticus 2.08% (n=1), S. hominis subsp. hominis 2.08% (n=1), S. hemolyticus 2.08% (n=1), and S. intermedius 2.08% (n=1). The latter species were identified at the species level using the RapID ONE System. The identification of S. aureus and S. epidermidis was validated by PCR employing species-specific primers (Figure 2).

Agarose gel at 1.2%. MWM: Molecular weight marker 1 kb Plus DNA Ladder (NEB, USA). Lane 1: Positive control for the nuc gene in S. aureus and S. epidermidis. Lanes 2–3: Positive samples for the nuc gene in S. aureus. Lane 4: Positive sample for the nuc gene in S. epidermidis.

**Azithromycin Resistance**

Among the 48 Staphylococcus strains analyzed, 47.92% (n=23) were resistant to azithromycin, with the following distribution: S. epidermidis 16.67% (n=8), S. aureus 20.83% (n=10), S. chromogenes 2.08% (n=1), S. saprophyticus 2.08% (n=1), S. hominis subsp. hominis 2.08% (n=1), S. hemolyticus 2.08% (n=1), and S. intermedius 2.08% (n=1). Additionally, 4.16% (n=2) of the S. aureus strains exhibited intermediate resistance.

**Characterization of Resistance Genes**

A total of 81.25% (n=39) of the isolates carried at least one of the resistance genes analyzed. The most prevalent gene was ermB, detected in 58.33% (n=28) of the positive samples, followed by ermT in 45.83% (n=22) and ermA in 6.25% (n=3). The mef(A/E) gene was not detected in any of the strains studied. The distribution of resistance genes by bacterial species is shown in Table 1.

**Table 1:** Distribution of ermA, ermB and ermT genes among Staphylococcus species. n = 53

Microorganism	ermA (3) %(n)	ermB (28) %(n)	ermT (22) %(n)
S. aureus	0	64.29 (18)	40.91 (9)
S. epidermidis	100 (3)	21.42 (6)	40.91 (9)
S. saprophyticus	0	3.57 (1)	0
S. hemolyticus	0	3.57 (1)	4.54 (1)
S. hominis ss. hominis	0	3.57 (1)	4.54 (1)
S. chromogenes	0	3.57 (1)	4.54 (1)
S. intermedius	0	0	4.54 (1)

The identified resistance genes were grouped into six patterns. The most prevalent pattern was ermB with 33.33% (n=16), followed by ermB–ermT with 20.83% (n=10) and ermT with 20.83% (n=10). The ermA–ermT, ermA–ermB, and ermA–ermB–ermT patterns were each found in 2.08% (n=1) of the samples. The distribution of these resistance patterns among the bacterial species identified is shown in Table 2.

**Table 2:** Resistance patterns identified in bacterial strains. n = 39

Microorganism	ermB % (n)	ermB–ermT % (n)	ermT % (n)	ermA–ermT % (n)	ermA–ermB % (n)	ermA–ermB–ermT % (n)
S. aureus	87.50 (14)	40.00 (4)	50.00 (5)	0	0	0
S. epidermidis	6.25 (1)	30.00 (3)	30.00 (3)	100 (1)	100 (1)	100 (1)
S. saprophyticus	6.25 (1)	0	10.00 (1)	0	0	0
S. hemolyticus	0	10.00 (1)	0	0	0	0
S. hominis subsp. hominis	0	10.00 (1)	0	0	0	0
S. chromogenes	0	10.00 (1)	0	0	0	0
S. intermedius	0	0	10.00 (1)	0	0	0
**Total**	100 (16)	100 (10)	100 (10)	100 (1)	100 (1)	100 (1)

The geographical assessment of macrolide resistance patterns in hospitalized COVID-19 patients revealed a distinct distribution of resistance profiles among Staphylococcus species by patients’ municipalities of residence. Among the most prominent findings, ermB was identified in three patients from Bucaramanga, and two from El Carmen de Chucurí; the ermB–ermT pattern was detected in Bucaramanga and Floridablanca; and ermT was mainly observed in Bucaramanga. Less common combinations were also found, such as ermA–ermT in San Gil and ermA–ermB–ermT in Puerto Wilches.

**Genotypic Resistance vs. Phenotypic Resistance**

The analysis of the association between genotypic and phenotypic resistance showed that 47.92% (n=23) of Staphylococcus species were resistant to azithromycin, while 39.58% (n=19) were resistant and carried at least one resistance gene (ermB and/or ermT). Among these, S. aureus and S. epidermidis accounted for 18.75% (n=9) and 12.50% (n=6), respectively, whereas Staphylococcus species represented 8.33% (n=4).

The presence of ermT was significantly associated with S. aureus and the presence of ermA with S. epidermidis (p < 0.05), with no association observed for other species isolated in the study (Table 3). Regarding the relationship between resistance genes and phenotypic resistance to azithromycin by species, S. aureus and ermT showed a statistically significant association (p < 0.05).

**Table 3:** Relationship between species and phenotypic and genotypic resistance. n= 19

Microorganism	Phenotypic Resistance % (n)	Genes					
		ermA % (n)	p-value	ermB % (n)	p-value	ermT % (n)	p-value
S. aureus	20.83 (10)	0	0.07a* 1a**	64.29 (18)	0.18b* 1a**	31.90 (7)	0.04b* 0.03a**
S. epidermidis	18.75 (8)	100 (3)	0.03a* 0.55a**	21.42 (6)	0.14b* 0.61a**	50.00 (11)	0.30b* 0.35a**

a: p-value from Fisher’s exact test. b: p-value from Pearson’s chi-square test. *: Relationship between bacterial species and macrolide resistance gene. **: Within-species relationship between phenotypic resistance and the macrolide resistance gene. P-values are reported at a significance level of less than 0.05; statistically significant values are shown in bold.

The amplified DNA fragments were successfully sequenced, yielding high-quality sequences with predominantly Q30 values, indicating an accuracy greater than 99.9%. The sequence lengths matched the expected sizes of the target genes after trimming low-quality bases at the ends. Forward and reverse reads were assembled using the CAP3 software, generating unique contigs for each fragment and their integrity was confirmed through manual inspection of the overlapping region. BLAST analyses of the assembled sequences confirmed the identity of the resistance genes ermB, ermT, and ermA. No relevant mutations or polymorphisms were observed in the analyzed sequences, indicating homogeneity in the detected resistance genes. These results provide molecular evidence of the presence and conservation of resistance genes in the studied strains.

## Discussion

This study demonstrated a high prevalence of macrolide-resistant S. aureus and S. epidermidis in hospitalized COVID-19 patients in Santander, with ermB and ermT being the most frequent genes. This finding is consistent with their known role in macrolide resistance among Gram-positive cocci, as they block the ribosomal target site and thereby limit therapeutic options for secondary infections in hospital settings^25^. The absence of a significant correlation between resistance genes and phenotypic resistance suggests the possible involvement of other mechanisms, such as the cMLSb and iMLSb phenotypes^26^, which justifies further studies to explore this relationship in greater depth.

A recognized limitation of the present study is the exclusive use of the agar diffusion method for assessing phenotypic resistance to azithromycin. Although standardized by CLSI M100-Ed34:2024^15^ and widely used in surveillance, this technique may underestimate certain resistance profiles that can be detected through quantitative methods such as microdilution. Nevertheless, since the objective of this study was not to compare techniques but rather to characterize resistance profiles within an epidemiological surveillance framework, priority was given to reproducible and feasible methods under real laboratory operating conditions. This methodological choice reflects the practical focus of the study; however, future research is recommended to complement these findings with reference methods that allow a more accurate evaluation of the minimum inhibitory concentration (MIC).

The extensive use of antibiotics during the COVID-19 pandemic has accelerated macrolide resistance worldwide^27^,^28^. The findings of the present study are comparable to those reported in Nigeria, where Bamigbola et al.^29^ documented a prevalence of S. aureus of 32.4% and S. saprophyticus of 31.5% in COVID-19-associated co-infections, and to those from Egypt, where Hamdy et al. ^30^ described a notable prevalence of S. aureus and S. epidermidis carrying ermB and ermT, although differing in the presence of mef(A/E), which was not detected in the samples analyzed in this study. This suggests a possible geographical variation in the distribution of these genes, influenced by species-specific characteristics and clinical environments.

In Latin America, although few studies are available, research conducted in Peru^11^,^31^ and Chile^12^ has reported a similar prevalence of resistance genes in Staphylococcus, with differences in the species and phenotypes involved. The absence of S. saprophyticus in this study, unlike in Nigeria^29^, is consistent with its primary association with urinary tract infections. These results underscore the importance of continuous surveillance in the region to characterize variations in resistance patterns and to tailor control strategies to each specific area.

Although S. epidermidis is generally not associated with respiratory infections, its presence in hospitalized COVID-19 patients suggests a possible opportunistic role in hospital-acquired co- infections. This observation highlights the importance of further investigating its role within the respiratory tract and its interaction with the host microbiota, particularly in immunocompromised patients or those undergoing prolonged treatments. Understanding this dynamic could reveal new risk factors in clinical settings and contribute to the development of more effective infection control strategies, considering the ability of S. epidermidis to adapt and persist in hospital environments^32^.

Although this study did not analyze detailed sociodemographic variables, such as social determinants of health or comorbidities, these factors could influence resistance patterns and should be considered in future research. Moreover, the lack of sufficient data to correlate resistance patterns with specific geographic locations (rural or urban) underscores the need to implement more robust antimicrobial surveillance systems in Santander to guide local interventions.

Despite being based on samples collected between 2020 and 2021, the results obtained in 2024 remain relevant, providing insight into how the pandemic and the intensive use of macrolides have influenced the development of resistance in the short- and medium-term. These data help identify resistance trends that may now be established, offering a foundation for formulating current policies on the rational use of antibiotics and serving as a reference for future studies in Colombia and the region.

The detection of erm genes in several municipalities suggests a widespread distribution of these resistance mechanisms, possibly driven by antibiotic use in both hospital and community settings. This underscores the need to view antimicrobial resistance as a regional issue that demands surveillance and control strategies tailored to local needs.

## Conclusions

This study provides evidence on the prevalence of S. aureus and S. epidermidis in hospitalized COVID-19 patients, highlighting their role as opportunistic pathogens in clinical settings. The identification of the macrolide resistance genes ermB and ermT underscores the growing concern about the increase in antimicrobial resistance, which could complicate infection management in vulnerable patients.

Although S. epidermidis is not typically associated with respiratory infections, its presence in this context suggests the need to investigate further its role in hospital co-infections and its interaction with the host microbiota. The high prevalence of these resistance genes emphasizes the urgency of implementing more controlled antibiotic use and effective infection control strategies within healthcare environments.
